# Ohio State Dental Society

**Published:** 1898-02

**Authors:** 


### Editorial.

### Ohio State Dental Society.

The regular annual meeting of this Society was held in Columbus, December 7th, 8th and 9th, with nearly all the active membership in attendance. The entire program was, with one slight exception, carried out to the full, and even that was compensated for by new matter introduced.

A number of excellent papers were read and fully discussed.

These will appear in the next two numbers of the REGISTER. The papers were interesting and instructive, bringing out new thoughts, and presenting some old ideas in new dress which is oftentimes as important as the more recent things.

A large share of one day was devoted to clinics, which many new and interesting processes were well described, fully demonstrated, to the satisfaction of the membership present.

Arrangements for the Tri-State meeting (which will be held in June next) elicited great interest. Many suggestions were made which will aid the committees having the matter in charge. Put-in-Bay was selected as the place of the next meeting of the Tri-State; quite a goodly majority voted in favor of this place.

There were twenty-five or thirty new names added to the list of membership. One very noticeable feature was the fact that more than ever the younger members took active part in the proceedings, and this is a hopeful sign, and ought to be encouraged. When the students that are now in the colleges will have entered the profession, and shall have entered upon society work, they will be far more active than the younger members have hitherto been. The training to which they are now subjected will fit them for such work better than heretofore. Indeed, the profession as a whole has never made more rapid progress than is now taking place.

